# Guiding TRAIL to cancer cells through Kv10.1 potassium channel overcomes resistance to doxorubicin

**DOI:** 10.1007/s00249-016-1149-7

**Published:** 2016-06-27

**Authors:** Franziska Hartung, Luis A. Pardo

**Affiliations:** Oncophysiology Group, Max-Planck Institute of Experimental Medicine, Hermann-Rein-Str. 3, 37075 Göttingen, Germany

**Keywords:** TRAIL, Kv10.1, Apoptosis, Chemotherapy resistance, Cancer therapy

## Abstract

Resisting cell death is one of the hallmarks of cancer, and represents a common problem resulting in ineffective cancer therapy. To overcome resistance to apoptosis, we designed an antibody-based therapy strategy using Kv10.1 as a target. Kv10.1 is a voltage-gated potassium channel, which has been identified as a tumor marker several years ago. The agent consists of a Kv10.1-specific single-chain antibody fused to the soluble tumor necrosis factor-related apoptosis-inducing ligand (scFv62-TRAIL). We combined scFv62-TRAIL with different chemotherapeutic drugs, all of which failed to induce apoptosis when used alone. In the combination, we could overcome the resistance and selectively induce apoptosis. Among the drugs, doxorubicin showed the most promising effect. Additionally, we observed improved efficacy by pre-treating the cells with doxorubicin before scFv62-TRAIL application. Expression analysis of the TRAIL death receptors suggests a doxorubicin-induced increase in the abundance of receptors as the mechanism for sensitization. Furthermore, we confirmed the anti-tumor effect and efficacy of our combination strategy in vivo in SCID mice bearing subcutaneous tumors. In conclusion, we propose a novel strategy to overcome resistance to chemotherapy in cancer cells. Doxorubicin and scFv62-TRAIL reciprocally sensitize the cells to each other, specifically in Kv10.1-positive tumor cells.

## Introduction

Resistance against chemotherapeutic agents is still a major obstacle for effective cancer therapy (Krishna and Mayer 2000), and the search for alternative therapeutic strategies and cancer-specific targets to efficiently treat cancer is stronger than ever (Volm and Efferth 2015). TRAIL, the tumor necrosis factor-related apoptosis-inducing ligand, is a promising candidate for cancer treatment and its soluble form is already in clinical trials, with limited success (Cheah et al. 2015). TRAIL is expressed on the surface of immune cells and binds to five different receptors. Two of them, TRAIL-R1 and TRAIL-R2, induce caspase activation and apoptotic cell death after ligand binding. TRAIL-R3 and TRAIL-R4 are decoy receptors, also expressed on the cell surface but lacking functional intracellular death domains (LeBlanc and Ashkenazi 2003). TRAIL-R5, also known as osteoprotegerin, is a soluble receptor and does not induce apoptosis (Emery et al. 1998). Compared to other death receptors, e.g., TNF or CD95, TRAIL is well tolerated, has low side effects and shows potent anti-tumor effect (Ashkenazi et al. 1999).

However, there are several generic problems using soluble TRAIL in therapy. Soluble TRAIL has a low in vivo half-life and less activation of TRAIL-R2, one of the two death receptors (Kelley et al. 2001; Muhlenbeck 2000). Therefore, relative high doses of active TRAIL are required and the selectivity for tumor cells may be lost. Additionally, TRAIL-R2 activity has been reported to increase invasiveness and metastatic potential of tumors (von Karstedt et al. 2015), making necessary a careful assessment of the therapeutic use of TRAIL.

We have previously reported the generation and initial characterization of a single-chain antibody-TRAIL fusion. The antibody targets residues 402–410 in the extracellular funnel of the pore of Kv10.1 (Gomez-Varela et al. 2007), a voltage-gated potassium channel that is very abundant in neurones, but also expressed by many non-neural tissues over a time window in G2/M phases of the cell cycle (Urrego et al. 2016). More than 70 % of tumor tissues and tumor cells from different origin express robustly the channel (Ding et al. 2007; Farias et al. 2004; Hemmerlein et al. 2006; Mello de Queiroz et al. 2006; Rodriguez-Rasgado et al. 2012). Downregulation using siRNA or blocking of the channel lead to growth inhibition of cancer cells in vitro and in vivo (Garcia-Ferreiro et al. 2004; Gomez-Varela et al. 2007; Weber et al. 2006); siRNA knockdown of Kv10.1 shows synergistic effects with TRAIL overexpression in osteosarcoma models (Wu et al. 2013). There is also evidence that anti-depressants able to block Kv10.1 improve survival in brain metastases moderately expressing Kv10.1 in human patients (Martinez et al. 2015). Therefore, cancer cells seem to take an advantage of expressing Kv10.1 and this cancer-restricted expression of the channel and the accessibility from the external environment turn the channel into a promising therapeutic target.

The cancer-selectivity and the potent apoptosis-inducing effect have been shown already in our previous study with prostate cancer cells (Hartung et al. 2011) in vitro. Here we extend and deepen our understanding on the mechanisms of sensitization, and provide the first evidence of efficacy in vivo using a xenograft tumor mouse model.

## Materials and methods

Unless otherwise indicated, reagents were obtained from Sigma-Aldrich (Munich, Germany).

### Cell culture

MDA-MB435S (ATCC HT29) was purchased from ATCC, and CHO-K1 (ACC-110) from DSMZ. MDA-MB435S cells were cultured in RPMI 1640 with 10 % FCS and CHO-K1 cells in Ham’s F-12 medium with 10 % FCS, or in protein-free expression medium Panserin C6000 at 37 °C in humidified 5 % CO_2_ atmosphere. Transfection of scFv62-TRAIL in CHO-K1 cells was done with Lipofectamine 2000 (Thermo Fisher Scientific) as recommended by the manufacturer. siRNA against Kv10.1 (Weber et al. 2006), TRAIL-R1 and/or TRAIL-R2 (both from Santa Cruz) was transfected using Lipofectamine RNAi Max (Thermo Fisher Scientific). The cells were treated with scFv62-TRAIL and Cycloheximide (CHX) 24 h after siRNA transfection and 20 h later apoptosis induction was analyzed.

### Production of scFv62-TRAIL

Supernatant from transfected CHO-K1 cell culture containing the scFv62-TRAIL was concentrated with Centricon YM-100 (Millipore), and sterile filtered. Purification was performed using affinity chromatography in a BioCAD Vision Workstation (Persective/Applied Biosystems). The affinity column was prepared using Self Pack POROS 20 EP coupled to the fusion protein used to generate the antibody (h1x) (Hemmerlein et al. 2006) Cell medium containing the scFv62-TRAIL construct was loaded on the column, washed with PBS pH 7.5 and eluted by acidic pH (100 mM Glycine/150 mM NaCl pH 2.4). The elution peak was collected in an automated fraction collector and neutralized with 1 M Tris–HCl pH 8.0.

Active scFv62-TRAIL concentration was determined based on the amount of TRAIL detected using a commercial ELISA (R&D Systems) according to the manufacturer protocol.

### Flow cytometry

Analysis of the apoptosis-inducing potential of scFv62-TRAIL was performed using Annexin V assays in a flow cytometer (BD FACS Aria). The cells were seeded at 1 × 10^5^ per well in 12-well plates and treated with various concentrations of scFv62-TRAIL in combination with 5 µg/ml (17.77 µM) CHX for the indicated times. Apoptotic cells were determined using the Annexin V-FITC/PI staining kit (Imgenex, San Diego, CA, USA) or Annexin-Alexa647 (Molecular Probes, Thermo Fisher Scientific) according to the manufacturer’s recommendations. Annexin V-positive cells were defined as apoptotic cells including early and late apoptotic cells.

### Surface plasmon resonance (SPR)

scFv62-TRAIL purified as above was dialyzed against HBS buffer, and protein concentration was determined using BCA protein assay (Thermo Fisher Scientific). The ligand used was h1x, coupled to a CM5 chip (GE Healthcare, Uppsala, Sweden) according to the instructions from the manufacturer at pH 4.5. Association and dissociation constants were determined in a BIAcore 2000 device. The concentration mentioned was calculated as total protein concentration assuming purity and the predicted molecular mass of 153 kDa. The fusion protein was diluted in buffer HBS-EP to concentrations ranging from 3.8 to 30.8 nM and injected on the flow cell. The BIAevaluation software was used for analysis and data were fitted to a 1:1 Langmuir model for determination of affinity, association, and dissociation constants.

### Quantitative real-time PCR

Total RNA was obtained from cells using the RNAeasy mini kit (Qiagen, Hilden, Germany) and first strand cDNA was produced using SuperScript (Thermo Fisher Scientific). Real-time PCR was performed with 100 ng cDNA using the TaqMan system in Light Cycler 480 (Roche Diagnostics). The housekeeping genes human transferrin receptor and human actin were used as a control of RNA integrity and for quantification. Specific mRNA content was determined using the Light Cycler 480 software. For all genes the UPL probe system from Roche Diagnostics was used.

### In vivo study

Animal studies were performed according to the German law and with the approval by the local animal ethics committee. We used 8-week-old female CB17/Icr-Prkdc^scid^/IcrCr mice (Charles River), housed in individually ventilated cages (two mice per cage), with free access to food (autoclaved pellets) and water. At the start of the experiment, mice were injected subcutaneously (s.c.) in the right side flank with 5 × 10^6^ MDA-MB435S cells in 200 µl PBS. The area of injection had been previously shaved and was maintained without hair to minimize measurement errors. Starting at 3 weeks, tumor size was controlled weekly by measuring the major axis (length) and a perpendicular diameter (width), using a caliper always by the same operator. Tumor volume was calculated with the following formula: tumor volume = length × width^2^ × 0.52. The animals were randomized after the first measurement point in four groups: (A) control, (B) doxorubicin (freshly prepared in PBS), (C) scFv62-TRAIL, and (D) doxorubicin in combination with scFv62-TRAIL. One treatment cycle lasted 4 days. On day 1, group A and C received 200 µl PBS and group B and C 200 µl doxorubicin in PBS (0.9 mg/kg). On the following 2 days, mice received scFv62-TRAIL (0.15 mg/kg) (group C and D) or PBS (group A and B). On day 4, mice were not treated. After three cycles, the dose of scFv62-TRAIL was reduced: On day 1, group A and C received 200 µl PBS and group B and C 200 µl PBS and doxorubicin (0.9 mg/kg). The following days, mice received scFv62-TRAIL (0.15 mg/kg) (group C and D) or PBS (group A and B). On days 3 and 4, mice were not treated. Tumor growth and body weight were measured once per week. Six weeks after tumor implantation, the animals were killed and the tumors were dissected and weighted.

### Statistical analysis

Data were analyzed using GraphPad Prism and are represented as mean ± SEM. At least two independent experiments were performed for each analysis. Statistical significance was evaluated by Student’s *t* test and two-way ANOVA.

## Results

### Combination of chemotherapeutic agents and scFv62-TRAIL can overcome resistance and induce apoptosis in MDA-MB435S cells

The construction and production of the Kv10.1-specific single-chain antibody fused to the soluble TRAIL (scFv62-TRAIL) has been described before. The scFv62-TRAIL was expressed in CHO-K1, concentrated and concentration was determined using a commercial TRAIL ELISA and is therefore reported as equivalent TRAIL concentration in the preparation. The expression yield was ~5 µg/ml. The selectivity for Kv10.1 expressing cancer cells and the potent apoptosis induction of our fusion construct was already demonstrated with prostate cancer cells.

**Fig. 1:**
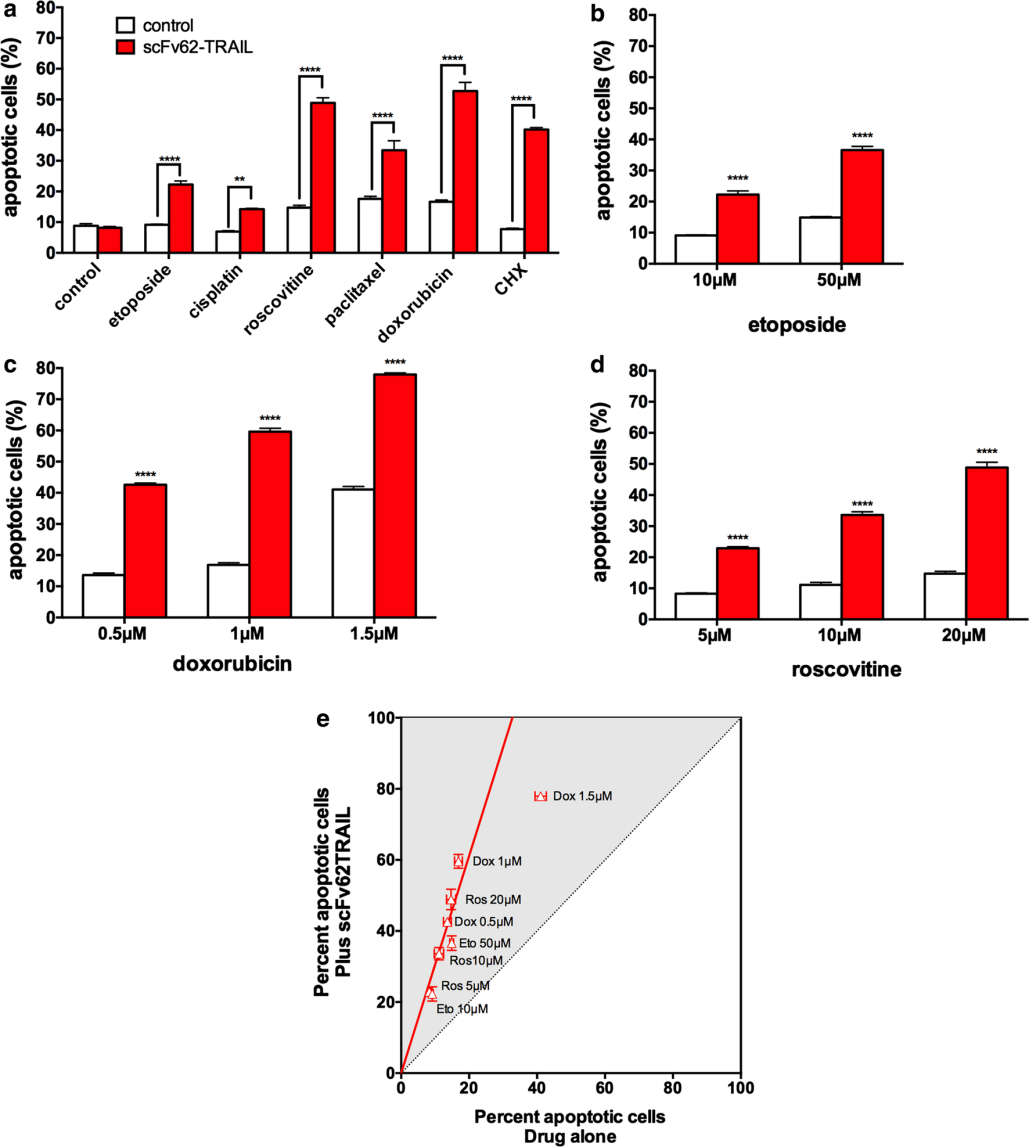
TRAIL-induced apoptosis in the presence of chemotherapeutic agents in MDA-MB435S cells. **a** MDA-MB435S cells were treated with the indicated chemotherapeutics (10 μM etoposide, 10 μM cisplatin, 20 μM roscovitine, 10 μM paclitaxel, 1 μM doxorubicin, 0.5 μg/ml CHX) in combination with scFv62-TRAIL (0.1 μg/ml) for 18 h. Apoptosis was determined by Annexin V staining and flow cytometry. *Asterisks* indicate statistical significance (**p < 0.01; ****p < 0.0001; two-way ANOVA). MDA-MB435S cells were treated with scFv62-TRAIL (0.1 μg/ml) with different concentrations of etoposide (**b**), doxorubicin (**c**), and roscovitine (**d**) and analyzed with Annexin V staining and flow cytometry. **e** Data from **b**–**d** are represented as percent apoptosis in the presence of scFv62-TRAIL versus apoptosis induction in the absence of the construct. The synergistic effect was comparable for all drugs and linearly correlated to the concentration of the drug

The highly metastatic and Kv10.1-positive cancer cell line MDA-MB435S is described to be resistant against many chemotherapeutic agents and also to TRAIL-induced apoptosis (Grosse-Wilde and Kemp 2008; Ortiz-Ferrón et al. 2008). We treated the MDA-MB435S cells with six conventionally used chemotherapeutic drugs in combination with scFv62-TRAIL (Fig. 1a). Treatment with scFv62-TRAIL alone did not induce a detectable increase of apoptotic cells. Only paclitaxel and doxorubicin induced significant levels of apoptotic cells when applied alone. The scFv62-TRAIL in combinational treatment increased significantly the amount of apoptotic cells for all tested agents. The most intense effect was observed with cycloheximide (CHX), roscovitine, and doxorubicin. Paclitaxel alone showed apoptosis induction of around 18 % and in combination with scFv62-TRAIL an apoptotic rate of 35 %. Also, CHX in combination with scFv62-TRAIL induced apoptosis in 40 % of the cells. scFv62-TRAIL in combination with cisplatin and etoposide induced a weak but statistically significant effect.

The sensitizing effect of scFv62-TRAIL to etoposide, doxorubicin, and roscovitine was dose-dependent. We treated the cells with different concentrations of the drugs alone or in combination (Fig. 1b–e). The scFv62-TRAIL increased the efficacy of all three drugs in all concentrations used in an almost linear fashion. The sensitization lost linearity only for the highest concentration of doxorubicin; the very high value of 80 % apoptotic cells could have saturated the detection capability of Annexin V.

### scFv62-TRAIL retains its affinity for the antigen

**Fig. 2:**
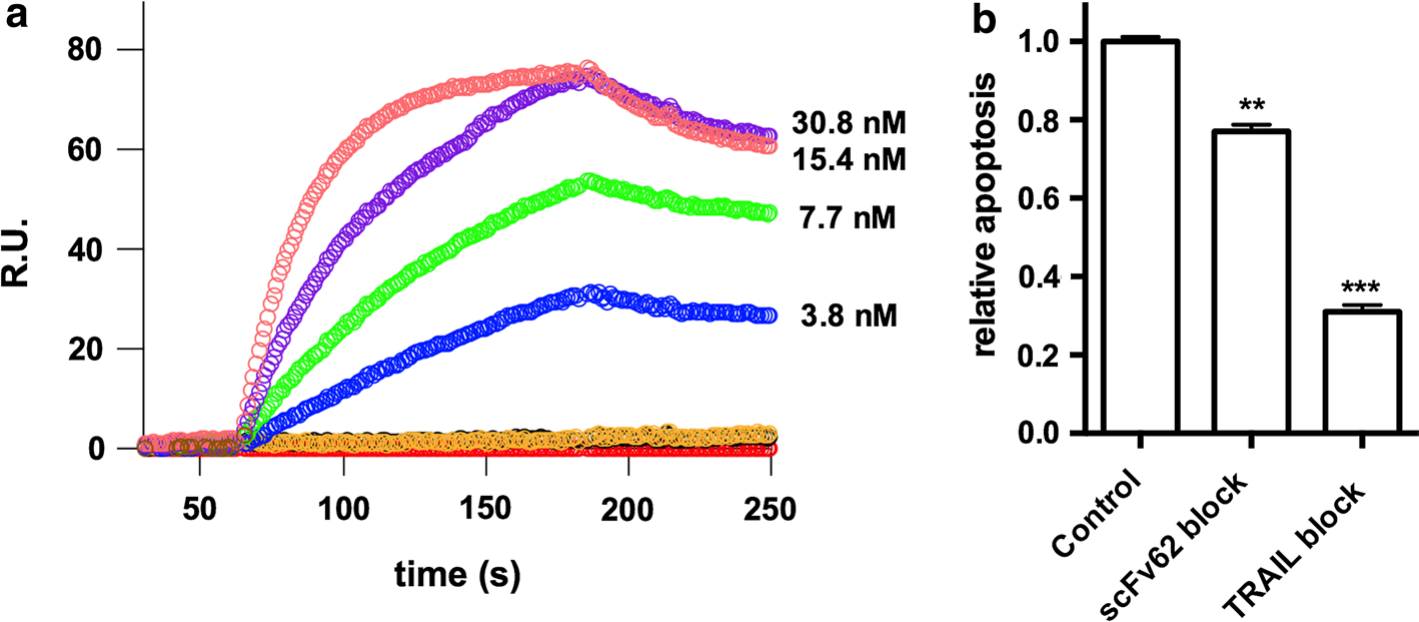
Analysis of scFv62-TRAIL induced apoptosis. **a** Association and dissociation constants between the immunogen (h1x) and the scFv62-TRAIL construct were determined by SPR. Sensograms were recorded for binding of the indicated concentrations of scFv62-TRAIL (analyte) to immobilized h1x (ligand). **b** Apoptosis induction by scFv62-TRAIL requires both the antibody part and active TRAIL. MDA-MB435S cells were treated with scFv62-TRAIL that had been pre-incubated with excess antigen (1:50) or with neutralizing anti-TRAIL antibody (1:10) for 1 h. Apoptosis induction was analyzed with Annexin V staining and flow cytometry 18 h after the addition 0.1 µg/ml scFv62-TRAIL and 0.5 μg/ml CHX. Student’s *t* test, **p < 0.01, ***p < 0.001

The single-chain antibody was cloned starting from a mouse hybridoma mAb62 (Hemmerlein et al. 2006) and fused to soluble TRAIL. To confirm that the antibody part still recognizes the antigen, we performed surface plasmon resonance (SPR) experiments (Fig. 2a). The fusion protein used as an antigen served as a ligand bound to the chip, and the scFv62-TRAIL construct was the analyte. The sensorgrams obtained with different concentrations of the analyte (3.8–30.8 nM) indicate an affinity of 0.67 nM. We are not in a situation to directly compare this value with the intact antibody or the single chain alone because the stoichiometry of the TRAIL fusion construct (trimer) is different from that of the antibody (dimer). However, we can nevertheless conclude that the scFv retains a reasonable affinity after fusion with TRAIL.

### Induction of apoptosis by scFv62-TRAIL requires both active TRAIL and a binding antibody moiety

To test if both active parts of the construct are required for its action, scFv62-TRAIL was pre-incubated with a neutralizing anti-TRAIL antibody (at a 10:1 ratio for 1 h) or with a fusion protein containing the epitope for scFv62 (h1x; 50:1 for 1 h), in order to block TRAIL—or the antibody binding site (Fig. 2b). MDA MB435S cells were subsequently treated with the preincubated construct in the presence of 5 µg/ml CHX for 18 h (Fig. 2b). Both the anti-TRAIL antibody and the antigen blocked the effect of the construct and resulted in significant reduction of apoptosis induction; blocking of TRAIL showed a stronger reduction of the efficacy. Therefore, we conclude that both moieties in scFv62-TRAIL are required to efficiently induce apoptosis.

### scFv62-TRAIL acts though both TRAIL-R1 and TRAIL-R2

**Fig. 3:**
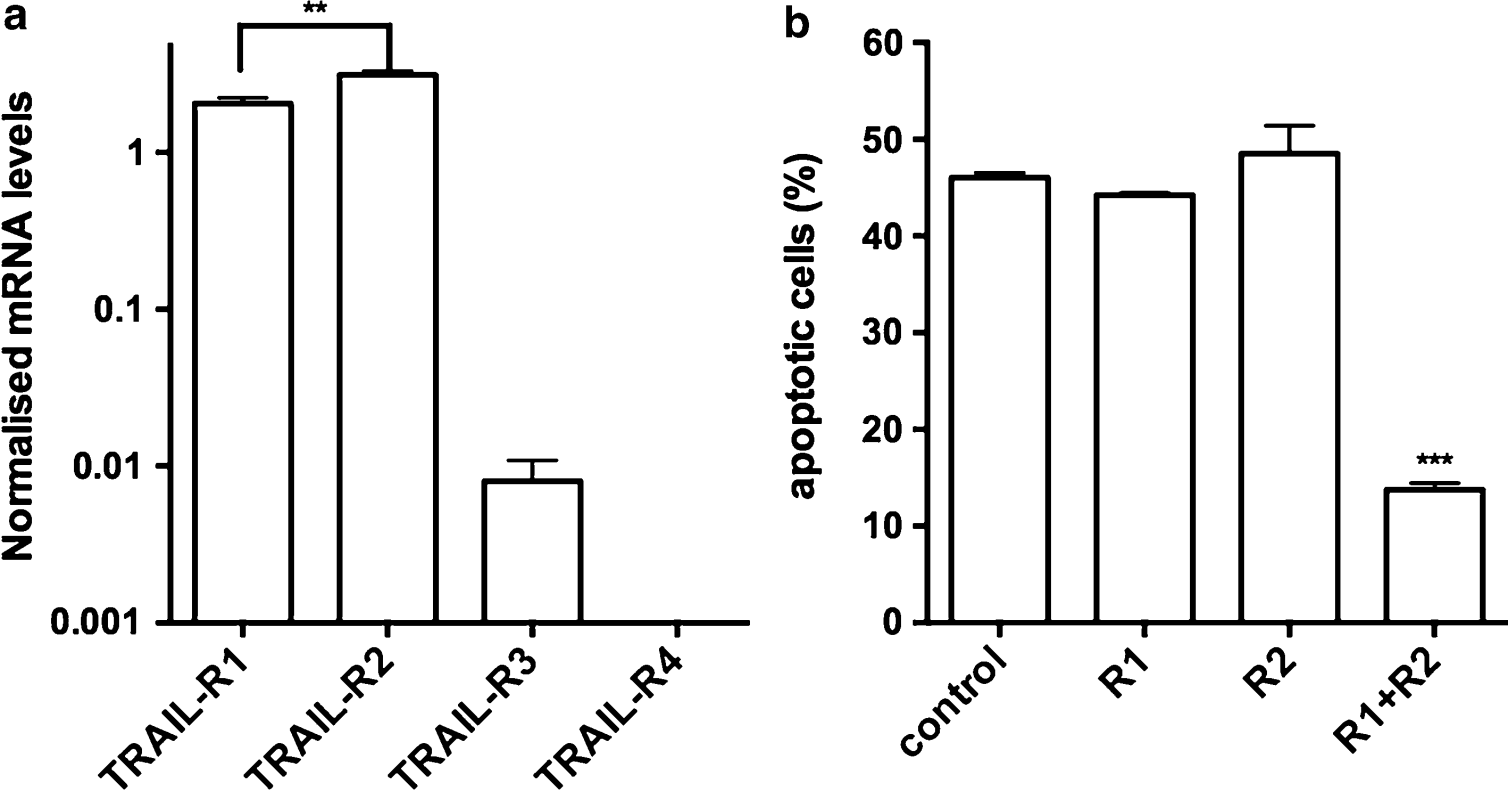
**a** Expression of TRAIL receptors mRNA in MDA-MB435S. Quantitative real-time PCR on cDNA from the cell line revealed abundant message for TRAIL-R1 and TRAIL-R2, the latter being more abundant. **b** Both TRAIL-R1 and TRAIL-R2 can mediate the effect to scFv62-TRAIL. MDA-MB435S cells were treated with siRNA against, TRAIL-R1, TRAIL-R2, or both apoptotic receptors in combination (30 nM); 24 h later cells were treated with scFv62-TRAIL (0.1 μg/ml) in combination with 0.5 μg/ml CHX for 18 h. Apoptosis induction was measured with Annexin V staining and flow cytometry. Only when both receptors were knocked one there was a significant reduction in apoptosis induction

There are two TRAIL receptors that mediate the apoptosis signal. Different studies demonstrated that TRAIL induces apoptosis predominantly via TRAIL-R1 in some cancer cell lines and via TRAIL-R2 in others. By real-time RT-PCR on mRNA obtained from MDA-MB435S cells, we found that both apoptosis inducing receptors (Kim et al. 2000) were expressed at significant levels, TRAIL-R2 being slightly more abundant than TRAIL-R1 (Fig. 3a). The levels of mRNA were normalized using both human transferrin receptor and human actin as invariably expressed genes. The decoy receptor TRAIL-R3 was scarce, and TRAIL-R4 was not detected.

To test which receptors are required for the action of scFv62-TRAIL, we knocked down the TRAIL apoptosis receptors with specific siRNAs before treatment with scFv62-TRAIL in combination with CHX (Fig. 3b). The presence of TRAIL-R1 or R2 was enough to maintain the efficacy of the construct. Only downregulation of both receptors together completely blocked the TRAIL-mediated apoptosis induction. Therefore, scFv62-TRAIL can induce apoptosis through either of the two death receptors.

### Pre-sensitizing effect of the chemotherapeutic agents

**Fig. 4:**
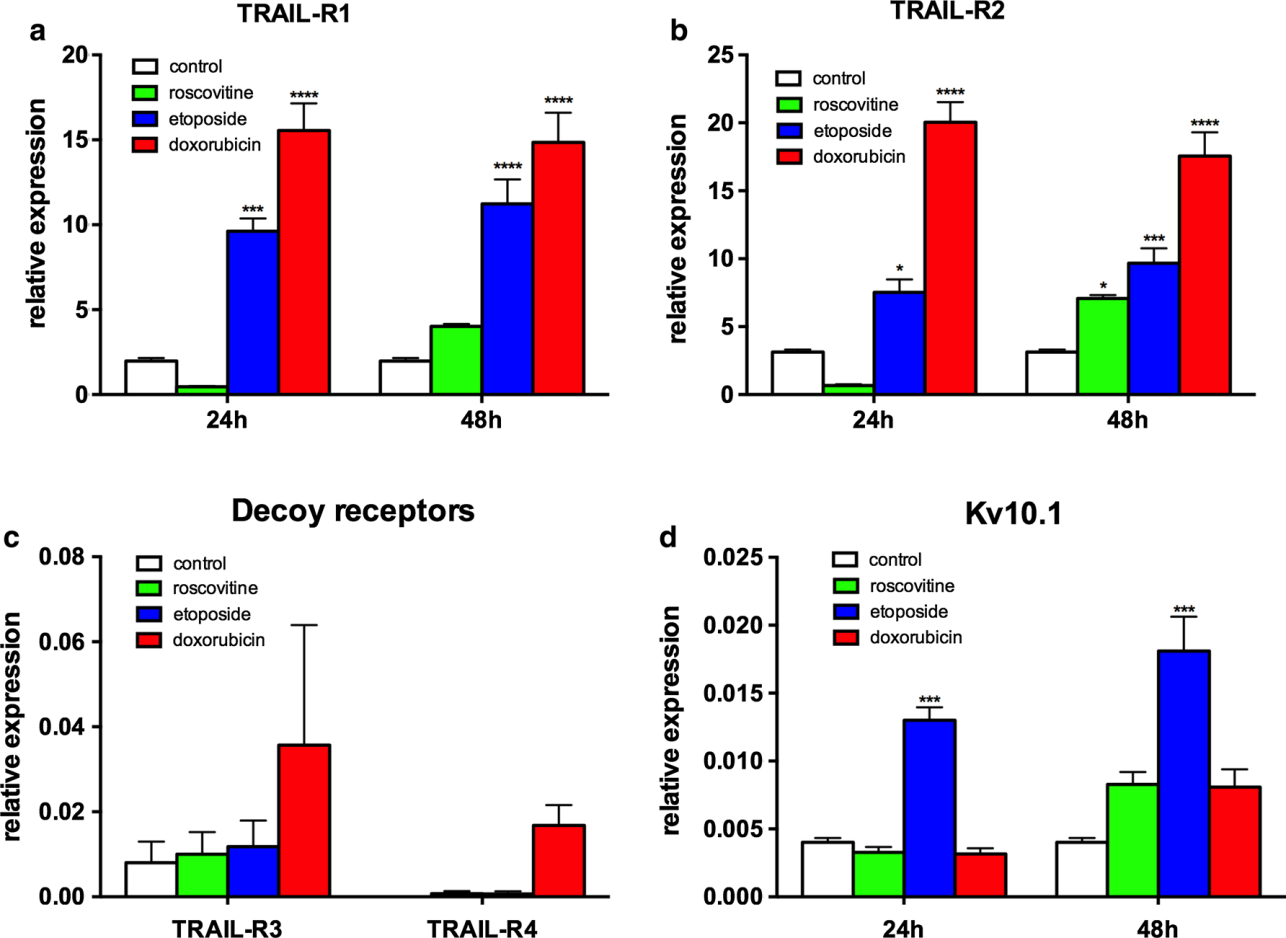
Influence of chemotherapeutic agents on expression of Kv10.1 and apoptotic receptors. MDA-MB435S cells were incubated for 24 and 48 h with 20 μM roscovitine, 50 μM etoposide, or 1.5 μM doxorubicin, and analyzed with quantitative real-time PCR for TRAIL-R1 (**a**), TRAIL-R2 (**b**), decoy receptors-R3 and -R4 at 24 h treatment (**c**), and Kv10.1 expression (**d**) (two-way ANOVA, ***p < 0.001; ****p < 0.0001)

Sensitizing with chemotherapeutic agents is a commonly used strategy to make cancer cells susceptible to TRAIL-induced apoptosis. The exact mechanism responsible for the sensitizing effect is not clear for every agent; there are many different steps in the apoptosis machinery that can be altered to become resistant against TRAIL. Since there was no or only poor expression of the decoy receptors, we focused on the expression rate of the apoptotic receptors (Fig. 4). We treated the MDA-MB435S cells with etoposide, doxorubicin, and roscovitine for 24 and 48 h and analyzed the mRNA abundance of TRAIL receptors and Kv10.1 by real-time PCR (Fig. 4). The amount of TRAIL-R1 and TRAIL-R2 was dramatically increased already after 24-h etoposide and doxorubicin treatment (Fig. 4a, b). Surprisingly, roscovitine induced a downregulation of TRAIL-R1 and TRAIL-R2 after 24 h and upregulation after 48 h treatment. TRAIL-R3 and TRAIL-R4 were virtually absent before and after treatment (Fig. 4c; note the different scale compared to a and b). Only etoposide induced a significant increase in Kv10.1 expression—up to 4 times—after treatment for 24 and 48 h (Fig. 4d). This could reflect the arrest in G2 typical of topoisomerase inhibitors (Clifford et al. 2003; Kolb et al. 2012; Wu et al. 2010), because Kv10.1 expression is maximal at this phase of the cell cycle (Urrego et al. 2016).

**Fig. 5:**
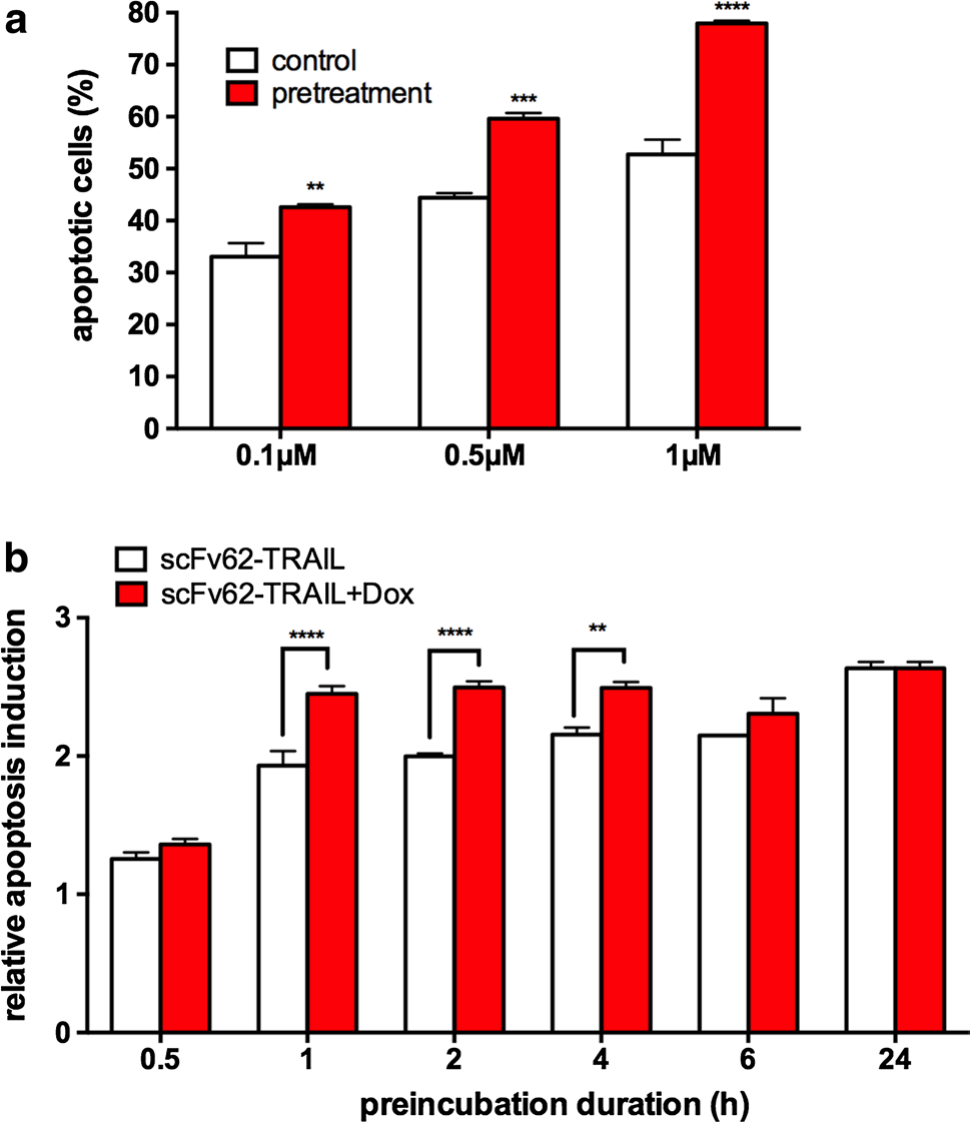
Pre-treatment increases apoptosis induction. **a** MDA-MB435S cells were treated with different concentrations of doxorubicin in combination with scFv62-TRAIL (0.1 μg/ml) for 18 h or pre-treated with doxorubicin for 24 h and then with scFv62-TRAIL (0.1 μg/ml) for 18 h. Afterwards, cells were analyzed with Annexin V staining and flow cytometry. **b** MDA-MB435S cells were treated with doxorubicin for 24 h and afterwards with scFv62-TRAIL for different time spans in presence (*red columns*) or absence of doxorubicin (*white columns*). After 18 h, apoptosis induction was measured using Annexin V staining and flow cytometry (two-way ANOVA, **p < 0.01; ***p < 0.001; ****p < 0.0001)

To explore the impact the changes in the expression of the apoptosis receptors and Kv10.1 on the efficacy of scFv62TRAIL, we pre-treated the MDA-MB435S cells with different concentrations of doxorubicin for 24 h and subsequently added scFv62-TRAIL for 18 h (Fig. 5a). Compared to the combinational treatment, we observed an increase of up to 25 % more apoptotic cells with the doxorubicin pre-treatment, which we attribute to the higher abundance of receptors.

Since we observed the highest apoptosis rate with doxorubicin, this agent was used for all following in vitro and in vivo experiments in combination with scFv62-TRAIL. To investigate the sensitizing effect of doxorubicin, we performed a pulse-treatment experiment. MDA-MB345S cells were treated with 0.5 μM doxorubicin for 24 h and afterwards with scFv62-TRAIL for different time spans in the presence or absence of doxorubicin (Fig. 5b). 1-h exposure to scFv62-TRAIL was already sufficient to induce the maximal apoptotic effect on MDA-MB435S cells, independently of the presence of doxorubicin. However, continued presence of doxorubicin increased the apoptosis induction at all times tested.

### Doxorubicin in combination with scFv62-TRAIL reduced tumor growth in vivo

**Fig. 6:**
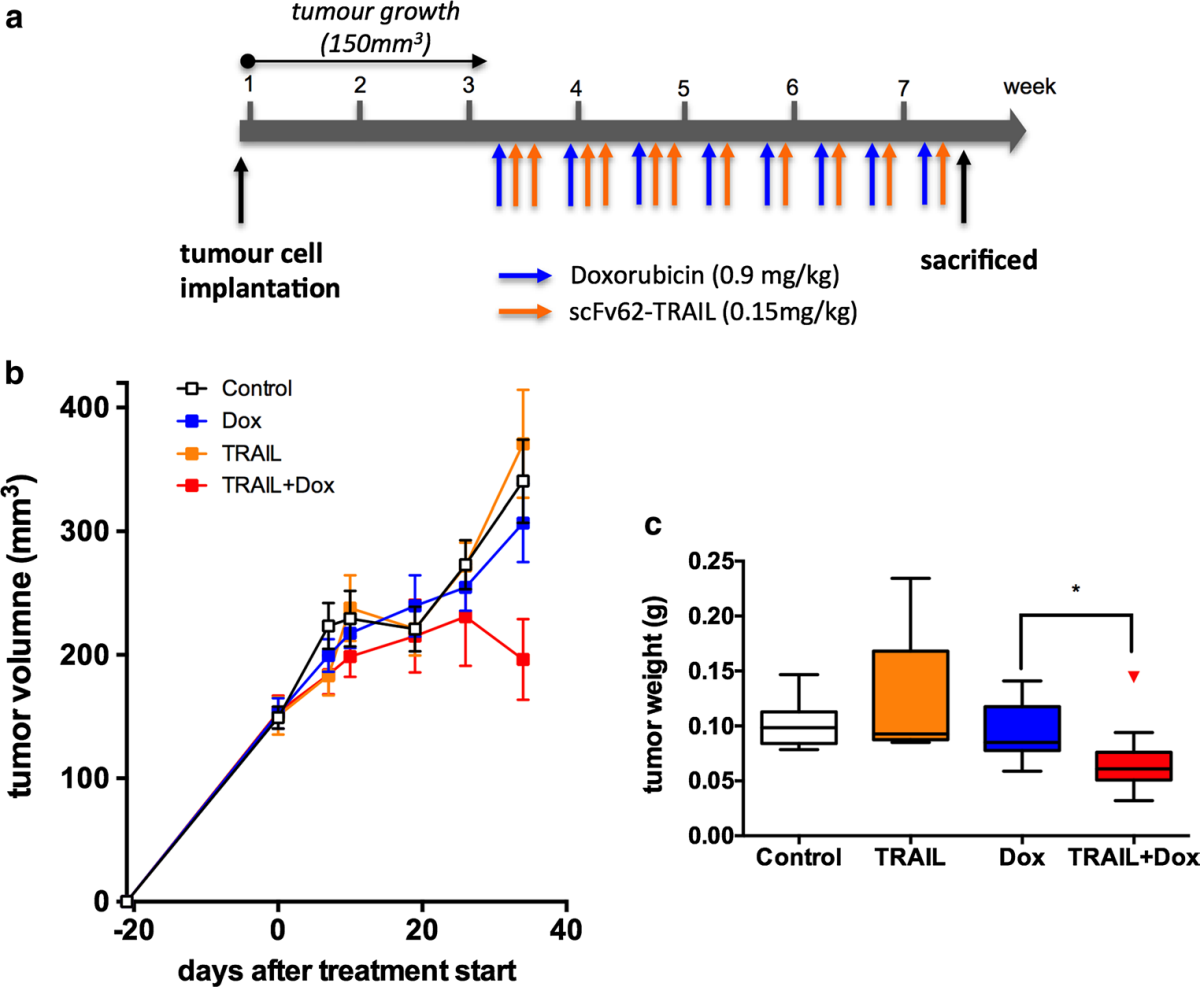
Efficacy of scFv62-TRAIL in vivo. 5 × 10^6^ MDA-MB435S cells were injected s.c. into SCID mice. After tumor development (150 mm^3^) mice we grouped and treated with doxorubicin (0.9 mg/kg), scFv62-TRAIL (0.15 mg/kg) or the combination of both (**a**). Tumor growth was measured once a week (**b**). **c** After six treatment cycles tumor weight was determined ex vivo (Kolmogorov–Smirnov test)

Based on the in vitro data, we performed an in vivo study with SCID mice carrying MDA-MB435S tumors. 5 × 10^6^ MDA-MB435S cells were injected s.c. into the flank of 8-week-old female CB17/Icr-Prkdc^scid^/IcrCr mice. When the tumors reached a measurable size (~150 mm^3^), the animals were randomized into four groups (12 animals per group) with similar means and standard deviation, and the treatment was started using intraperitoneal injections of doxorubicin (0.9 mg/kg) on day 1, and scFv62-TRAIL (0.15 mg/kg) on days 2 and 3. This loading cycle was repeated three times, allowing 1 day between cycles. Subsequently, doxorubicin and scFv62-TRAIL were administered on days 1 and 2, respectively, and days 3 and 4 the mice were not treated (Fig. 6a). In the corresponding control groups, the omitted agent was substituted by an equal volume of the vehicle (PBS). After six treatment cycles, a strong decrease of tumor growth was detected in mice treated with a combination of doxorubicin and scFv62-TRAIL (Fig. 6b). Ex vivo analysis of the tumor weight confirmed a significant reduction of the tumors in the mice that received the combinational treatment compared to the other groups (Fig. 6c).

## Discussion

A growing body of evidence demonstrates the involvement of ion channels in the hallmarks of cancer (Hanahan and Weinberg 2011; Prevarskaya et al. 2010). Among ion channels, the voltage-gated potassium channel Kv10.1 has been studied intensely (Ouadid-Ahidouch et al. 2016; Pardo and Stuhmer 2014) in this context.

The idea of targeting Kv10.1 for therapy is not new. Channel blockers (e.g., Astemizole) and a specific blocking antibody decrease tumor cell proliferation in vitro and in vivo (de Guadalupe Chavez-Lopez et al. 2015; Downie et al. 2008; Garcia-Quiroz et al. 2014; Gomez-Varela et al. 2007). However, for an effective tumor therapy it is not sufficient just to reduce the cancer cell proliferation. The cancer cells need to be eliminated to induce tumor regression. We designed a therapeutic fusion construct consisting of a Kv10.1-specific single-chain antibody and the apoptosis-inducing ligand TRAIL. TRAIL has been shown to be effective in eliminating cancer cells by inducing apoptosis and by fusing the ligand to a Kv10.1-specific antibody can overcome the limitations of the soluble TRAIL, like short half-life or less specificity (Hartung et al. 2011). In addition, resistance against TRAIL-induced apoptosis is a common phenomenon in cancer cells and is part of the immune escape strategy. The generation of a single-chain antibody TRAIL fusion offers several advantages. Compared to whole antibodies, single-chain antibodies are smaller and less immunogenic with a higher penetration rate, which is important for solid tumors (Yokota et al. 1992).

Roscovitine, etoposide, and doxorubicin are commonly used chemotherapeutic agents in cancer therapy (Baldwin and Osheroff 2005; Ortiz-Ferrón et al. 2008; Rivankar 2014), but the development of resistance, especially in metastatic stages, remains a major obstacle of an effective tumor treatment (Wang et al. 2014).

We chose for our in vivo study the Kv10.1-positive metastatic cancer cell line MDA-MB435S, despite its disputed origin (Lacroix 2009; Nerlich and Bachmeier 2013), because this model responds relatively well to anti-Kv10.1 therapy and this was important for a proof-of-concept study. We used then low doses of a chemotherapeutic (doxorubicin) and increased the response to treatment using a scFv-TRAIL fusion.

To overcome the multidrug resistance in MDA-MB435S, we used scFv62-TRAIL in combination with etoposide, roscovitine, and doxorubicin, and in all cases we observed an increase in apoptotic cells. A wide range of chemotherapeutic drugs has been tested in combination with TRAIL (Gill et al. 2007; Ortiz-Ferrón et al. 2008; Shamimi-Noori et al. 2008; Singh et al. 2005; Wang et al. 2010). The anthracycline antibiotic doxorubicin showed the highest apoptosis induction in the MDA-MB435S cells in combination with scFv62-TRAIL. TRAIL-mediated apoptosis is known to be increased in combination with doxorubicin (Komdeur et al. 2004; Voelkel-Johnson et al. 2002; Wang et al. 2010), while resistance to both agents in isolation is also reported (Xu et al. 2014; Zang et al. 2014).

To explore the mechanism underlying induction of apoptosis in combinational therapy, we blocked the TRAIL or the antibody part of our construct before treating the MDA-MB435S cells in the presence of doxorubicin; both approaches induced a decrease in apoptotic cells. Block of the TRAIL moiety was more efficient than block of the antibody, arguably because the construct can still act as soluble TRAIL in the absence of membrane binding though Kv10.1.

The requirement of TRAIL activity was also shown by siRNA-mediated downregulation of the TRAIL death receptors. We observed no change in the apoptosis induction, when only one receptor is downregulated, and only the inhibition of the expression of both receptors blocked the apoptosis induction. Therefore, our scFv62-TRAIL antibody can induce, in combination with chemotherapy, apoptosis via both death receptors in MDA-MB435S cells.

The MDA-MB435S cells are not sensitive to our scFv62-TRAIL construct or soluble TRAIL alone. There are several cellular changes that can lead to TRAIL resistance. One highly discussed factor is the expression level and composition of proapoptotic and decoy receptors (Riccioni et al. 2005; Sanlioglu et al. 2005). TRAIL-R1 and TRAIL-R2 are expressed in MDA-MB435S cells. Both death receptors are upregulated in the MDA-MB435S cells after sensitizing with doxorubicin and etoposide (Fig. 4), suggesting involvement of death receptor expression levels in TRAIL resistance of this cell line. The decoy receptors do not seem to contribute to TRAIL resistance in MDA-MB435S cells since TRAIL-R3 is weakly expressed and TRAIL-R4 is undetectable. Other studies found no correlation between decoy receptor expression and resistance to TRAIL in various cancer cell types (Mahalingam et al. 2009).

Upregulation of death receptors can be an explanation for the increase in apoptotic induction upon pre-treatment with chemotherapeutics (Fig. 4), because the TRAIL receptors would be more abundant in the cells before TRAIL treatment. Upregulation of TRAIL receptors R1 and R2 upon activation of p53 and/or NF-κB as a result of subtoxic doses of doxorubicin, etoposide, and other chemotherapeutic drugs are well documented (Gibson et al. 2000; Kang et al. 2005; Lee et al. 2002). Whether or not this is actually the mechanism responsible for the upregulation observed here has not been studied. Decoy receptors TRAIL-R3 and -R4 can also be upregulated by p53 (Meng et al. 2000), but the levels detected in our cell lines are only marginal even after chemotherapeutic drug treatment, and therefore it is unlikely that they play a role in inducing resistance.

The increase in Kv10.1 levels observed with etoposide alone could be due to arrest in G2 phase of the cell cycle, which is the moment when expression of Kv10.1 peaks in other cell types (Urrego et al. 2016).

scFv62-TRAIL in combination with doxorubicin also showed efficacy in vivo. We used a relative low amount of 0.9 mg/kg doxorubicin and 0.15 mg/kg scFv62-TRAIL per mouse in comparison with other studies (Vitovski et al. 2012; Wang et al. 2010), but the effect was still clearly observable. We noticed no evidence of increased aggressiveness of the tumors in the treated animals (von Karstedt et al. 2015). Nevertheless, experiments in mice with an intact immune systems would be required to progress along this line.

As a general conclusion, we provide here the in vivo proof of concept of the use of scFv62-TRAIL to overcome resistance to doxorubicin in Kv10.1-positive tumors.
